# Evaluation of structural changes in orbitofrontal cortex in relation to medication overuse in migraine patients: a diffusion tensor imaging study

**DOI:** 10.1590/0004-282X-ANP-2020-0360

**Published:** 2021-06-23

**Authors:** Aygul Tantik Pak, Sebahat Nacar Dogan, Yildizhan Sengul

**Affiliations:** 1 Gaziosmanpasa Training and Research Hospital Department of Neurology Istanbul Turkey Gaziosmanpasa Training and Research Hospital, Department of Neurology, Istanbul, Turkey.; 2 Gaziosmanpasa Training and Research Hospital Department of Radiology Istanbul Turkey Gaziosmanpasa Training and Research Hospital, Department of Radiology, Istanbul, Turkey.

**Keywords:** Migraine Disorders, Medication Overuse Headache, Orbitofrontal Cortex, Diffusion Tensor Imaging, Transtornos de Enxaqueca, Cefaleia por Uso Excessivo de Medicamentos, Córtex Pré-Frontal, Imagem por Tensor de Difusão

## Abstract

**Background:**

Migraine is a prevalent neurological disease that leads to severe headaches. Moreover, it is the commonest among the primary headaches that cause medication overuse headache (MOH). The orbitofrontal cortex (OFC) is one of the structures most associated with medication overuse.

**Objective:**

To determine microstructural changes in the OFC among migraine patients who developed MOH, through the diffusion tensor imaging (DTI) technique.

**Methods:**

Fifty-eight patients who had been diagnosed with migraine based on the Classification of Headache Disorders (ICHD-III-B) were included in the study. Patients were sub-classified into two groups, with and without MOH, based on the MOH criteria of ICHD-III-B. DTI was applied to each patient. The OFC fractional anisotropy (FA), and apparent diffusion coefficient (ADC) values of the two groups were compared.

**Results:**

The mean age of all the patients was 35.98±7.92 years (range: 18-65), and 84.5% (n=49) of them were female. The two groups, with MOH (n=25) and without (n=33), were alike in terms of age, gender, family history, migraine with or without aura and duration of illness. It was found that there was a significant difference in FA values of the left OFC between the two groups (0.32±0.01 *versus* 0.29±0.01; p=0.04).

**Conclusions:**

An association was found between MOH and changes to OFC microstructure. Determination of neuropathology and factors associated with medication overuse among migraine patients is crucial in terms of identifying the at-risk patient population and improving proper treatment strategies specific to these patients.

## RESUMO

**Introdução:**

A migrânea é uma doença neurológica prevalente que causa fortes dores de cabeça. Além disso, é a mais comum entre as cefaleias primárias que causam cefaleia por uso excessivo de medicamentos (CUEM). O córtex orbitofrontal (OF) é uma das estruturas mais associadas ao uso excessivo de medicamentos.

**Objetivo:**

Determinar alterações microestruturais no córtex OF em pacientes com migrânea que desenvolveram CUEM, por meio da técnica de imagem por tensor de difusão (ITD).

**Métodos:**

Cinquenta e oito pacientes com diagnóstico de migrânea, com base na Classificação das Cefaleias (ICHD-III-B), foram incluídos no estudo. Os pacientes foram subclassificados em dois grupos, com e sem CUEM, com base nos critérios de CUEM da ICHD-III-B. A ITD foi aplicada a cada paciente. Os valores de anisotropia fracionada OFC (AF) e coeficiente de difusão aparente (CDA) dos dois grupos foram comparados.

**Resultados:**

A média de idade de todos os pacientes foi de 35,98±7,92 anos (variação: 18‒65), sendo 84,5% (n=49) do sexo feminino. Os dois grupos, com CUEM (n=25) e sem (n=33), são semelhantes em termos de idade, sexo, história familiar, migrânea com ou sem aura e duração da doença. Verificou-se que houve diferença significativa nos valores de AF do córtex OF esquerdo entre os dois grupos (0,32±0,01 *versus* 0,29±0,01; p=0,04).

**Conclusões:**

Foi encontrada associação entre o CUEM e as alterações na microestrutura do córtex OF. A determinação da neuropatologia e dos fatores associados ao uso excessivo de medicamentos entre pacientes com migrânea é crucial para identificar a população de pacientes em risco e melhorar as estratégias de tratamento adequadas específicas para esses pacientes.

## INTRODUCTION

Migraine is the most common primary headache impacting the population of patients who are at a younger and more productive age^1^. Comorbid conditions that occur with severe debilitating headaches lead to serious social and economic burdens. Neuropsychiatric symptoms such as anxiety (particularly panic and phobia), depression, bipolar disorder, obsessive-compulsive disorder and nicotine dependence, along with psychiatric disorders such as substance abuse^2^^,^^3^, frequently accompany migraine^3^.

Medication overuse headache (MOH) occurs mostly among patients who have chronic migraines. It has been observed that MOH develops in 8.2% of the migraine patients living in Brazil^4^. MOH recurs in nearly 30% of the patients within one year following discontinuation of pain medication and adjustment of the treatment^5^. Given this knowledge and studies that have assessed the association between migraine and substance abuse, it is considered that there is a predisposition link between migraine and substance abuse^3^.

The basic features of substance abuse, such as compulsive drug use and drug-induced recurrence, have been found to be partially caused by changes in the functioning of the orbitofrontal cortex (OFC)^6^^,^^7^. Through hypofunctioning of the OFC, the inhibition mechanism is impaired^8^, and this induces an increase in impulsivity and deterioration in reward and decision-making mechanisms^6^^,^^9^. This is observed among substance abusers and contributes to development of MOH in migraine patients. In a PET study, glucose metabolism was measured before and after drug withdrawal in chronic migraine and MOH cases, and several regions of the brain that are associated with pain were found to be hypometabolic. However, they were rapidly reactivated upon withdrawal of analgesics. On the contrary, it has been observed that hypometabolism in the OFC remained despite drug withdrawal, and it has been put forward that this might be associated with relapses among MOH patients^10^. Given this information, we established the hypothesis of our research on the premise that MOH could develop as a result of behavioral pathologies induced by structural impairments in the OFC of migraine patients.

Diffusion tensor imaging (DTI) is a magnetic resonance imaging (MRI) technique that is used to map and characterize the three-dimensional diffusion of water as a function of spatial location. Several DTI parameters are used to assess diffusion and, indirectly, fiber tract microstructure. DTI shows where neuronal/axonal loss occurs as a result of neurodegeneration and inflammation. Fractional anisotropy (FA) measures the anisotropic diffusion of water molecules and the average diffusion coefficient (ADC) describes the magnitude of the average molecular displacement through diffusion^11^. In the literature, only a limited number of neuroimaging studies have been conducted among migraine patients who developed MOH, and there^10^^,^^12^^,^^13^ is no study that has assessed the OFC through DTI.

In our research, we aimed to compare the OFC of patients with and without MOH, through the method of region of interest (ROI) based on DTI.

## METHODS

### Study procedure

Our research was designed as a prospective, observational cross-sectional study. The study was performed in accordance with the ethical guidelines that were stated in the “Helsinki Declaration” and was approved by the Gaziosmanpasa Training and Research Hospital Ethics Committee. Written informed consent was obtained from the participants after they had been given precise explanations about the scope of the procedures.

### Participants

Eighty-two patients who had been admitted to the neurology outpatient clinic and diagnosed with migraine were included in the study. The inclusion criteria were that the participants needed to be between the ages of 18‒65 and to be diagnosed with migraine based on the International Classification of Headache Disorders (ICHD-III-B). This classification states that migraine is a unilateral throbbing severe headache that lasts for 4 to 72 hours; it is described as a primary headache that arises due to physical activity and co-occurs with nausea, vomiting, phonophobia and photophobia^14^. Our exclusion criteria comprised presence of the following: additional headaches (apart from migraine and MOH), neurodegenerative diseases, history of neurosurgery procedure, head trauma, history of stroke, previously known psychiatric disorders, withdrawal from the study, use of a prosthesis that was incompatible with MRI, use of metal appendages (metal kneecaps and pacemakers), claustrophobia and cerebral lesions that hindered examination of cranial MR imaging of the patient (including lacunar infarctions and leukoaraiosis, detection of vascular lesions in cranial MRI or poor quality of MRI). As a result, 24 patients excluded. Thirteen patients with migraine declined to participate or did not go to their MRI appointment. After the MRI, 11 additional patients were excluded due to leukoencephalopathy. Fifty-eight patients completed the study protocol and these formed our sample for analysis.

The sociodemographic characteristics of the patients, family history, duration of illness, presence of aura, frequency of monthly episodes and the names and quantities of analgesics that were being administered to the patients every month were recorded. To measure the severity of pain, a user-friendly visual analogue scale (VAS)^15^ was used, in which the “0” point represented the absence of any pain, while the “10” point represented the most severe pain in the patient's life.

The patients were subdivided into two groups: those who had MOH and those who did not. In accordance with the International Classification of Headache Diseases (ICHD-III-B), MOH was defined as a situation in which a patient who has had primary headache on more than 15 days/month for more than three months has drug intake on more than 10 days a month over a period of at least 3 months in the case of ergotamine, triptans, opioids and combinations of analgesics in particular; while for simple analgesics this situation was considered to consist of regular intake of drugs on more than 15 days a month over a period of at least 3 months^14^. All of our participants were using simple analgesics at the time of the interview.

### Magnetic resonance imaging protocol

**Figure 1:**
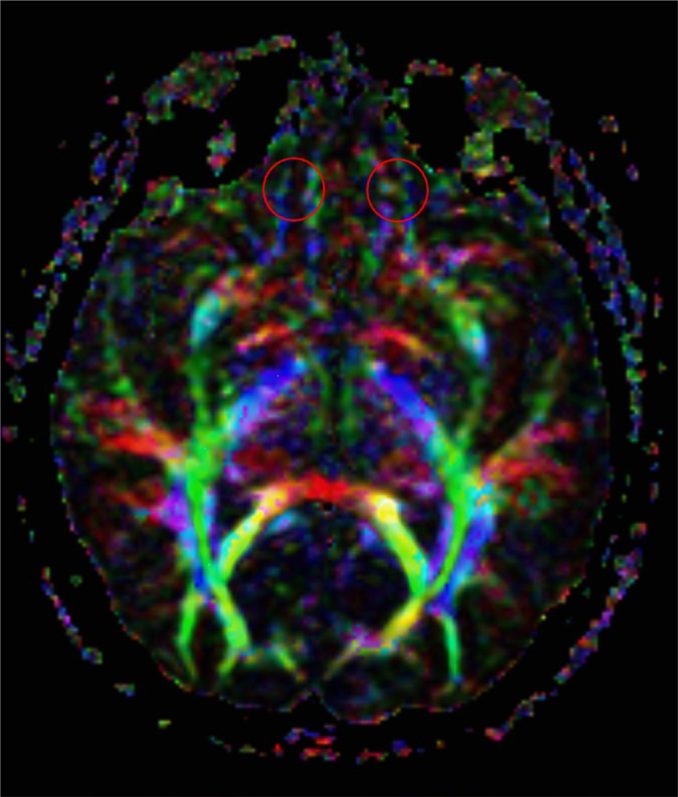
Placement of the regions of interest in the orbitofrontal region.

MRI was performed using 1.5 T-MRI units (GE Signa Explorer; GE, Milwaukee, WI, USA). 3D T1W volumetric sequences (TR/TE/TI, 8.7/3.2/450 ms) without contrast were applied using fast gradient brain volume imaging (BRAVO) with an isotropic voxel resolution of 1 mm. Generalized parallel imaging was applied by using auto-calibrating reconstruction for cartesian (ARC) with an acceleration factor of two for phase-encoding direction. The DTI included a single-shot, spin-echo, echo-planar sequence with TR: 4950 ms TE: 102 ms; matrix: 128 × 128 field of view: 230 mm and slice thickness 5.5 mm; and 24 diffusion-encoding directions were used with the values of b=0 s/mm^2^ and b=1000 s/mm^2^. Parallel imaging was performed through ARC with an acceleration factor of two. The Advantage Workstation (AW) scanner console (software version 4.6; GE Healthcare) was used for fractional anisotropy (FA) apparent diffusion coefficient (ADC) map reconstruction. The 3D T1W images were used as anatomical references for placement and tracing of ROIs. These images were coupled with the corresponding region of FA-ADC maps at the same section level. All the ROIs were drawn manually in circular shapes with constant size. The adaptation of the sizes and placement of the ROIs in the OFC (Figure 1) were achieved through simultaneous assessment by experienced radiologists (SND). The radiologist was blinded to neurological symptoms during the imaging analysis.

### Statistical analysis

The IBM SPSS statistics software, version 20.0, was used for the statistical analysis of the data. Categorical measurements were evaluated as numbers and percentages, while numerical measurements were evaluated as the mean and standard deviation (or median and minimum-maximum), and descriptive statistical methods were used. The distribution of the data was evaluated based on the Shapiro-Wilk test. In cross-group comparisons, the independent-sample t test was used for data with normal distribution, whereas the Mann-Whitney U test was used for data that did not have normal distribution. The patients were subdivided into two groups (with and without MOH). The OFC DTI metrics of the two groups were compared. To assess the association between OFC and MOH (dependent variable: DTI value of OFC; independent variables: age, gender, duration of illness, frequency of the episodes, pain severity and presence of MOH), multivariate regression analysis was used. Statistical significance was considered as p<0.05 in all tests.

## RESULTS

The mean age of all the patients was 35.98±7.92 years (range: 18-65), and 84.5% (n=49) of them were female, while 15.5% (n=9) of them were male. Among all the patients, 67.24% (n=39) had a family history of migraine; and 39.7% (n=23) had migraine with aura, while 60.3% (n=35) had migraine without aura. The mean duration of the disease was 7.36±7.26 years, while the incidence of migraines per month was 8.13±4.90, and the mean VAS value was 8.87±1.20. It was determined that the mean OFC FA values of all patients were 0.29±0.05 × 10^-3^ mm^2^/s on the right side and 0.30±0.05 × 10^-3^ mm^2^/s on the left side; while the mean values of OFC ADC was 8.27±0.61 × 10^-3^ mm^2^/s on the right side and 8.14±0.54 × 10^-3^ mm^2^/s on the left side. The amount of analgesic, which was administered in all patients, was 11.50±10.27 per month and 43.1% (n=25) of the patients had MOH (Table 1).

**Table 1:** Sociodemographic, clinical and diffusion tensor imaging data of all the patients.

Age (mean±SD)		35.98±7.92 years
(min-max values)		(18‒51)
Gender % (n)		
	Female	84.5% (n=49)
	Male	15.5% (n=9)
Family history % (n)		
	Yes	67.24% (n=39)
	No	42.86% (n=19)
Aura % (n)		
	Yes	39.7% (n=23)
	No	60.3% (n=35)
Duration of diagnosis of migraine (mean±SD)		7.36±7.26 years
Monthly incidence of migraine (mean±SD)		8.13±4.90
VAS score (mean±SD)		8.87±1.20
OFC FA (mean±SD)		
	Right	0.29±0.05 × 10^-3^ mm^2^/s
	Left	0.30±0.05 × 10^-3^ mm^2^/s
OFC ADC (mean±SD)		
	Right	8.27±0.61 × 10^-3^ mm^2^/s
	Left	8.14±0.54 × 10^3^ mm^2^/s
Medication overuse headache % (n)		
	Yes	43.1% (n=25)
	No	56.9% (n=33)

SD: standard deviation; VAS: visual analog scale; OFC: orbitofrontal cortex; FA: fractional anisotropy; ADC: apparent diffusion coefficient.

The patients were divided into two groups: those with MOH (group I) and those without MOH (group II). The two groups were similar in terms of age, gender, family history, migraine with aura/without aura and duration of diagnosis of migraine (p>0.05). It was found that there was a significant difference in FA values of the left-side OFC between the two groups (0.32±0.01 *versus* 0.29±0.01; p=0.04) (Table 2).

**Table 2:** Comparison of sociodemographic, clinical and diffusion tensor imaging data of the groups.

		MOH (+) (n=25)	MOH (-) (n=33)	p-value
Age (mean±SD)		36.80±1.60	35.36±1.37	0.50*
(min-max)		(18‒51)	(18‒49)	
Gender % (n)				
	Female	88.00% (n=22)	81.82% (n=27)	
	Male	12.00% (n=3)	18.18% (n=6)	0.71**
Family history % (n)				
	Yes	68.00% (n=17)	66.67% (n=22)	
	No	32.00% (n=8)	33.33% (n=11)	0.97**
Aura % (n)				
	Yes	40.00% (n=10)	39.39% (n=13)	
	No	60.00% (n=15)	60.61% (n=20)	0.74**
Duration of diagnosis of migraine (mean±SD)		6.68±1.07	7.87±1.47	0.51*
Monthly incidence of migraine (mean±SD)		11.48±0.88	5.60±0.62	<0.001*
Amount of painkiller administered per month (pcs/month)		19.60±2.14	5.36±0.61	<0.001*
VAS score (mean±SD)		9.20±0.17	8.63±0.24	0.06*
OFC FA (mean±SD) x 10^-3^ mm^2^/s				
	Right	0.30±0.01	0.29±0.01	0.71*
	Left	0.32±0.01	0.29±0.01	0.04*
OFC ADC (mean±SD) x 10^-3^ mm^2^/s				
	Right	8.30±0.61	8.23±0.60	0.69*
	Left	8.20±0.55	8.05±0.52	0.29*

MOH: medication overuse headache; SD: standard deviation;

* independent-sample t test;

** Pearson's chi-square; OFC: orbitofrontal cortex; FA: fractional anisotropy; ADC: apparent diffusion coefficient.

The multivariate regression analysis, which was performed to assess the relationship between the presence of MOH and left OFC FA value, independent of age, gender, duration of illness, incidence of the attack and severity of pain, detected that there was a significant correlation between the MOH and the FA value of the left OFC (ß=0.43; p=0.01).

## DISCUSSION

In our study, we found a significant relationship between occurrence of MOH and changes to the OFC in patients who had been diagnosed with migraine, which was also compatible with our hypothesis. Our findings suggest that medication overuse behavior might be a consequence of a susceptibility to substance abuse due to OFC impairment. However, it should be kept in mind that medication overuse may also cause OFC impairment. This is a bidirectional relationship. The OFC plays a vital role in generating and using outcome predictions.

In a study evaluating chronic migraine patients (n=42) with medication overuse, using neuropsychiatric tests, these patients manifested significant deterioration in orbitofrontal task performance, compared with patients with episodic migraine (n=42) and a control group without headache (n=41)^16^. In another study evaluating medication overuse among migraine patients through neuropsychological tests, Iowa gambling task scores for OFC function were assessed. It was found that there was a significant deterioration in decision-making tests and Iowa gambling task scores among migraine patients with addictive-like behavior, whose decision-making and outcome perception was impaired despite the adverse impacts of medication overuse^17^. Taking into consideration these findings, we aimed to evaluate the microstructure of the OFC by means of the DTI MRI technique, among migraine patients who developed MOH. Consequently, microstructural impairment, which we detected in the OFC, accounts for the finding that our migraine patients in whom MOH developed displayed behavior of continuing to take medication even though they were aware of the potential detrimental impacts of medication overuse.

It has been revealed in studies evaluating MOH patients through the voxel-based morphometry MRI technique that the OFC volume was smaller in non-responding MOH patients^18^^,^^19^. In a study by Riederer et al., the gray matter volumes of patients with MOH were measured by using voxel-based morphometry MRI. Whereas increases in the volumes of the thalamus bilaterally and the ventral striatum were observed, there were decreases in the volumes of the OFC, anterior cingulate cortex, insula and precuneus^20^. In a study on MOH through functional MRI, it was revealed that the functioning of the primary somatosensory cortex, inferior parietal lobule, supramarginal gyrus and regions of the lateral pathway of the pain matrix returned to normal six months after discontinuation of the painkillers that the patients had been receiving. Based on this finding, it was suggested that MOH did not cause irreversible damage^21^. However, the regions that were examined through MRI in that study were merely the regions that play a role in the pain mechanism. It was also reported in another study, in which positron emission tomography was used, that recovery from hypometabolism occurred in these regions following discontinuation of the medication. Nonetheless, the hypometabolism that had been detected in the OFCs remained after cessation of the medication and MOH relapse was linked to the OFC damage^10^. This finding is also compatible with those of previous studies, in which it was suggested that changes to the OFC were associated with impaired decision-making ability and behavior of drug and substance abuse^22^^,^^23^. Similarly, in our study, the microstructural changes to the OFC in migraine patients were found to be compatible with MOH. However, in our study, the microstructure of the OFC was assessed through DTI MRI, and this was done using a novel design, compared with the previous functional MRI and PET studies.

Our research and other studies in the literature relating to the OFC demonstrated that impairment of the patients’ management of behaviors occurs. These outcomes can be foreseen, as conclusions drawn from the functional, structural and volume changes in the OFC. It could be considered that migraine patients’ pathological drug use behavior develops in MOH as a result of an impairment in the OFCs of these patients. Indeed, the fact that changes to the OFC were found to be significantly associated with FA values based on the DTI findings from patients with MOH verifies this argument.

### Limitations

Only the OFC was assessed in our study, and the linkages of the OFC, limbic and paralimbic regions were not assessed. Following cessation of these patients’ medications, their MRIs were not repeated. Moreover, no psychiatric interviews or neuropsychological tests on OFC function (objection alternation and object reversal learning tasks, gambling tasks, go/no-go tasks, olfactory recognition, theory of mind and social processing measures, and self-rating or family-rating scales on the patient's behavior)^24^ were conducted on our patients to determine their behavioral pathologies.

In conclusion, the OFC is a cortical structure that enables individuals to adjust and control their behaviors and to benefit from the consequences, through conclusions that they have drawn from the events that they experienced previously, for the new circumstances that they encounter.

Our research gives rise to the notion that frequent medication overuse among migraine patients could be associated with dysfunction of this region. This result is crucial since it indicates that the OFC could be a marker for response to treatment or could be a manifestation of adverse outcomes. Furthermore, from our review of the literature, we determined that there were no studies analogous to ours, in terms of study design. We believe that, thanks to this finding, we have made a remarkable contribution to identification of the pathophysiology of MOH. However, in future studies, it might be pertinent to identify whether the process is reversible, through repeating patients’ MRIs and investigating whether there would be a continuation of impact on the OFC, after regulation of these patients’ prophylactic treatments and cessation of their analgesics.
